# Realizing Racial and Ethnic Neighborhood Preferences? Exploring the Mismatches Between What People Want, Where They Search, and Where They Live

**DOI:** 10.1007/s11113-015-9369-6

**Published:** 2015-06-19

**Authors:** Esther Havekes, Michael Bader, Maria Krysan

**Affiliations:** Department of Sociologie/Interuniversity Center for Social Science Theory and Methodology, Utrecht University, Padualaan 14, 3584 CH Utrecht, The Netherlands; Department of Sociology, American University, 4400 Massachusetts Avenue, NW, Washington, DC 20016 USA; Department of Sociology/Institute of Government and Public Affairs, University of Illinois at Chicago, 1007 West Harrison Street (MC 312), Chicago, IL 60607-7140 USA

**Keywords:** Racial residential preferences, Housing search, Residential segregation, Chicago

## Abstract

The housing search process is an overlooked mechanism in the scholarly research that seeks to understand the causes of persistent racial residential segregation in the United States. Past research has explored in detail the preferences people hold in terms of the racial and ethnic composition of their neighborhoods, and more recently some have also examined the correspondence between racial and ethnic neighborhood preferences and current neighborhood racial/ethnic composition. But an intermediate stage—the racial/ethnic composition of where people search—has not been investigated. We analyze a subsample (*n* = 382) from the 2004–2005 Chicago Area Study to demonstrate the value of systematically studying the matches—or mismatches—between preferences, search locations, and neighborhood outcomes. We find that for whites, not only their current neighborhoods but also the neighborhoods in which they search for housing have larger percentages of whites than they say they prefer. In contrast, blacks—and to a lesser extent Latinos—search in neighborhoods that correspond to their preferences, but reside in neighborhoods with a larger percentage own group. Logistic regression analyses reveal that mismatches are associated with both a lack of information and inadequate finances, but also may be due to socially desirable responding for whites in particular. Our results provide suggestive evidence of the importance of unpacking the search process more generally and draw attention to what are likely to be productive new future data collection efforts as well as an area potentially ripe for policy interventions.

## Introduction

Racial and ethnic residential segregation remains high for blacks and is growing for Latinos in the United States (Timberlake and Iceland 2007; Logan and Stults 2011). Racial residential preferences are one of the three main explanations offered by scholars seeking to understand continued racial residential segregation in the United States (e.g., Clark 1992; Schelling 1971). Although some work demonstrates that residents prefer to live among co-ethnics (e.g., Charles 2006), it is also the case that many Americans express a desire for some racial and ethnic diversity in their neighborhoods. Latinos and African Americans, however, express this desire more than whites (e.g., Charles 2006; Farley et al. 1994). At the same time, most Americans live in neighborhoods with mostly people of their own race (Crowder et al. 2012; Iceland et al. 2002). One possible reason for this mismatch is that people answering survey questions about neighborhood racial preferences feel a need to provide interviewers with socially desirable answers. People might want to think of themselves as tolerant and over-state their true desire for diversity (e.g., Krysan 1998; Krysan and Couper 2003). They therefore actually search for housing, this argument goes, in neighborhoods with less diversity than they say they prefer.

Alternatively, people might honestly desire the level of diversity they report to interviewers but that desire for diversity is outweighed by other factors in the housing search. That is, people may need to choose between neighborhoods based on the neighborhood’s racial diversity, ease of travel to jobs, school quality, and affordability (among other neighborhood features), and they might value the latter neighborhood characteristics more than its racial diversity. Relatedly, with comparatively few integrated neighborhoods in any given metropolitan area, the possibility of finding a neighborhood with the desired level of integration might be relatively small. This is complicated by the fact that racial composition tends to correlate with other neighborhood characteristics (e.g., Krysan et al. 2009), making it even harder to satisfy all criteria in the housing search. The tension between stated preferences (those that respondents tell interviewers) and revealed preferences (those preferences inferred by people’s actions) has been thoroughly debated in the literature (e.g., Vasanen 2012).

But missing from this debate is that both of these explanations rely on the assumption that the ultimate move residents make reflects the outcome of choices made *during a search process.* The housing search process—and especially its racial and ethnic dimensions—is something that is largely unstudied. The social desirability explanation would maintain that people do not search for housing in areas as diverse as they say they want. The revealed preferences literature would suggest that people do search for housing in diverse neighborhoods but compromise on their preference for diversity for other neighborhood amenities. Regardless of which of these interpretations is correct, what is clear is that the *process* that links the stated preferences and revealed preferences—the actual housing search—has not been studied systematically. The purpose of this analysis is to begin to do just that. We use the most detailed data available on expressed racial preferences and housing searches, combine it with theoretically grounded expectations about why preferences, searches, and outcomes may be (mis)matched, and thereby provide an exploratory analysis intended to lay the groundwork for future data collection and theorizing about housing searches and their role in shaping neighborhood racial patterns.^1^

Specifically, to demonstrate the value of focusing on the search process, we use the 2004–2005 Chicago Area Study (CAS). For reasons we will delineate, these data are not ideal; however, this is the most complete data available which include residents’ stated racial residential preferences, retrospective residential search histories in 41 communities in the Chicago metropolitan area, and current residential location, thus enabling us to examine how racial and ethnic composition between these three types of communities (where people prefer, where they search, and where they live) might differ. In doing so, we begin to shed light on the search process as an overlooked mechanism that can help explain the perpetuation of racial residential segregation nearly a half-century after the end of the Civil Rights Movement and the passage of the Fair Housing Act. Drawing on spatial assimilation, place stratification, and housing information theories, we explore the possible direction of (mis)matches between the racial and ethnic composition of where people say they would ideally like to live, where they searched for housing, and where they currently live. Two main research questions guide our analysis: (1) Do whites, blacks, and Latinos search for housing and reside in neighborhoods they say they prefer in terms of the neighborhood’s racial and ethnic composition and (2) what factors contribute to a mismatch between preferences, the racial/ethnic composition of search locations and the current neighborhood?

## Previous Research

Many studies investigate racial and ethnic residential preferences, with most of them using a show card method in which respondents evaluate hypothetical neighborhoods that show varying degrees of racial and ethnic diversity (e.g., see Farley et al. 1994, 1978). This research demonstrates that an individual’s race is an important factor in shaping neighborhood preferences. Whites tend to favor predominantly white neighborhoods, while blacks prefer integrated neighborhoods (i.e., a 50 % white, 50 % black neighborhood) and see ‘all white’ but also ‘all black’ neighborhoods as least desirable (Bobo and Hutchings 1996; Clark 1991; Farley et al. 1994; Krysan and Farley 2002). For their part, Latinos favor a 50 % Latino, 50 % white neighborhood over an ‘all Latino’ neighborhood (Charles 2005). Because the original show card method created only a limited set of racial compositions and because it showed only two groups at a time (Farley et al. 1978), Charles (2000) created a different survey instrument in which respondents were asked to create their ideal neighborhood composition. Using this tool, Charles (2006) found that all groups drew quite integrated neighborhoods but also had a preference for at least a substantial number of co-ethnics that exceeded the share of any other outgroup. This was true for all groups, but especially for whites.

More recently, studies seek to uncover the factors that underlie these ethnic neighborhood preferences. Vignette experiments, in which respondents were presented with hypothetical neighborhoods in which relevant neighborhood characteristics systematically varied, have been successfully used to separate ethnic/racial motivations from socioeconomic concerns. These studies indicate that part of the racial preference patterns can indeed be attributed to neighborhood concerns that are *related* to race, such as poverty or crime (Emerson et al. 2001; Krysan et al. 2009; Lewis et al. 2011; St. John and Bates 1990). However, race itself remains an important factor in shaping residential preferences.

Studies have begun to investigate how these stated racial residential preferences correspond to the actual neighborhood racial composition of an individual. Clark (1992) demonstrated that although most blacks preferred an integrated black–white neighborhood, only 7 % realized this preference. Among whites and Latinos, most realized their preference for either a predominantly white (for whites) or predominantly Latino (for Latinos) neighborhood. Importantly, however, of those who preferred an integrated neighborhood, a large majority still moved to a neighborhood that was predominantly inhabited by their own group.

Freeman (2000) examined the role of racial/ethnic preferences in residential attainment processes, while taking into consideration household income, wealth, and other background characteristics. He showed that black and Latino preferences for living with whites were positively related to the percentage white in the respondent’s actual neighborhood. Similarly, Ihlanfeldt and Scafidi (2002) concluded that blacks with a stronger preference for black neighborhoods are also more likely to reside in such neighborhoods, though they maintained that preferences played only a minor role in residential attainment. Finally, Adelman (2005) demonstrated that preferences do not perfectly correspond with actual neighborhoods. His study in Atlanta, Boston, Detroit, and Los Angeles showed that middle-class blacks who prefer to live in a racially integrated neighborhood live on average in neighborhoods that are 60 % black and 30 % white, while middle-class whites who prefer racially integrated neighborhoods reside in predominantly white neighborhoods (i.e., on average 85 % white).

Overall, the above studies showed that—although residential preferences correlate with actual compositions—generally respondents live in neighborhoods with more co-ethnics than they report preferring. The purpose of this study is to draw attention to the intermediate stage between preferences and outcomes—namely, the housing search process itself—as a possible mechanism for understanding how this (mis)match occurs. Treating the housing search as the intermediate stage between what people say they want and what they have, we can imagine at least two ways a mismatch can occur. A mismatch between preferences and current neighborhood composition could occur because people might not search in neighborhoods that match their preferences (henceforth, we refer to this as Stage 1) or they might search in preferred neighborhoods but end up living in neighborhoods that do not match the search locations (i.e., Stage 2).

To date, there are only a few studies that examine the role of racial composition in housing searches. Krysan (2008) found that in the Detroit metropolitan area whites mainly search in all white communities, whereas blacks explore more varied types of neighborhoods, ranging from predominantly white to predominantly black. Relatedly, Krysan and Bader (2007) investigated the Detroit-area neighborhoods people would “seriously consider” and “never consider.” Whites mainly consider predominantly white neighborhoods as serious options, while blacks would seriously consider neighborhoods with large as well as small percentages of blacks. Bader and Krysan (2015) replicate their research in the Chicago area and find that the patterns of whites’ racial matching and blacks’ openness to communities of many racial compositions. They find that Latinos were open to many communities, but search patterns emulated white preferences after controlling for economic factors.

In the present study, we examine the neighborhood preferences of whites, blacks, and Latinos and their relationship to the racial and ethnic composition of neighborhoods in which they searched for housing and in which they currently live. Although we are highlighting a new way to think about the causes of racial residential segregation—by focusing on the housing search process—we draw on existing theories of spatial assimilation, place stratification, and housing information to inform our understanding of why mismatches might occur and how housing searches contribute to them. The existing models emphasize constraints during the process of neighborhood selection that result in a mismatch between preferences and outcomes. Yet, the nature of the constraints highlighted by these theories differs and they are likely to operate at different stages of the process. In the following, we discuss these theories and elaborate on who is likely to have a mismatch, in what stage, and in which direction.^2^

## Who is Likely to Have a Mismatch, at What Stage, and in Which Direction?

The *spatial assimilation model* (Alba and Logan 1993; Massey 1985) argues that the ability of individuals to realize their residential desires is a function of a household’s opportunities and constraints—particularly socioeconomic. Socioeconomic resources (such as income and education) increase a household’s available choice of alternatives in the housing market (De Groot et al. 2011) so that mismatches between preferences and outcomes may be due to inadequate socioeconomic resources to actualize preferences. Applying this model to our research, (a lack of) socioeconomic resources might create a mismatch at either stage. First, people may search in neighborhoods similar to their preferences, but those places might turn out to be financially unattainable (Stage 2). Additionally, households might consider their financial possibilities when making a first selection of possible neighborhoods to search and, hence, a lack of socioeconomic resources might also prevent people from searching in neighborhoods that match their preferences (Stage 1).

We expect the role of socioeconomic resources in shaping the direction of mismatches to function differently for whites, blacks, and Latinos, because of the relationship between neighborhood socioeconomic characteristics and neighborhood racial/ethnic composition. The racial and ethnic composition of neighborhoods is strongly related to its socioeconomic status. Neighborhoods with substantial African American and Latino populations (as compared to white populations) are generally associated with socioeconomic disadvantage and poverty (e.g., Charles 2003; Krivo et al. 2009). Consequently, a lack of socioeconomic resources for blacks and Latinos would likely result in more ‘own group’ members in their neighborhood than they prefer, while for whites, fewer resources will result in fewer ‘own group’ members than they prefer.

The *place stratification model* (Alba and Logan 1993; Massey 1985) emphasizes institutional barriers that have their origin outside of the household as the primary driver of segregation. This model expects racial and ethnic minority groups to be less likely to realize their residential preferences because of discrimination. Housing discrimination could influence the housing search by generating mismatches at both stages. First, a person might search in a particular neighborhood, but be deterred from living thereby discriminatory actions by real estate agents, landlords, or loan officers. This would create a mismatch between the search locations and outcomes (Stage 2). Given patterns of discriminatory treatment, this would be most likely to affect African Americans and Latinos.

But geographic steering, a less direct form of discrimination that continues to be uncovered by audit studies (Turner and Ross 2005) is another way that place stratification theories can shed light on the housing search process. Specifically, geographic steering is the idea that different racial and ethnic groups are shown different kinds of neighborhoods during the search process. This likely decreases the opportunity to search and reside in neighborhoods that match racial and ethnic preferences. Since geographic steering takes place when people are searching for a place to live, it may be especially influential on the neighborhoods in which people search for housing (and, thus, constitute a mismatch in Stage 1). The direction of steering and its impact on mismatches varies depending on the race and ethnicity of the searcher. Specifically, whites are encouraged to consider predominantly white and more affluent neighborhoods, whereas blacks and Latinos are steered to a more restricted number of less affluent minority neighborhoods (Farley 1996; Turner and Ross 2005). In this way, processes of geographical steering might lead to whites, blacks, and Latinos both searching and subsequently residing in neighborhoods that have a larger percentage of their own group than they prefer.

Finally, *housing information theories* emphasize a factor that is largely neglected in traditional spatial assimilation and place stratification arguments, i.e., knowledge of housing options, which may influence the mismatch between preferences and search locations in particular (i.e., Stage 1). This fits in the general category of the cognitive resources used in housing searches (Özüekren and Van Kempen 2002), but for our purposes, we focus specifically on knowledge of the communities in which one might search (e.g., see Krysan and Bader 2009). Specifically, insufficient knowledge of the housing market may prevent people from searching in neighborhoods that match their preferences, resulting in a mismatch between residential preferences and actual spatial patterns. To successfully find housing in neighborhoods that meet the wishes of residents in terms of racial/ethnic composition, residents need at least some knowledge about the possible communities in their search area that fit their preferences (Brown and Moore 1970; Krysan and Bader 2009). It is unlikely (though not impossible) that people search in communities they know nothing about, or at least the search costs of gathering information about these communities are higher (Krysan and Bader 2009). Additionally, knowledge of the specific neighborhoods in an area has an important impact on where people eventually end up living (Clark 1986).

To the extent that knowledge differs for racial/ethnic groups and to the extent that knowledge varies depending on the racial/ethnic composition of the neighborhood, then this knowledge can help us to understand who experiences mismatches in the search process, and in which direction. Krysan and Bader (2009) showed, for example, that Latinos have less general knowledge about different communities in the Chicago metropolitan area and African Americans’ generally less knowledge about overwhelmingly white and distant suburbs might complicate the search for housing in more integrated neighborhoods. Whites, on the other hand, are less aware of racially mixed—even those that are diverse but still majority white—neighborhoods. Hence, whites, blacks, and Latinos alike have better knowledge of neighborhoods dominated by their own racial/ethnic group and this might lead to searches (and moves) to neighborhoods that have larger percentages of their own group than they would prefer.

Finally, to the degree that individual searches are informed by social networks, we may find that individuals both search and reside in neighborhoods with greater percentages of their own group because these likely race-homogenous networks provide information that is similarly racially homogenous. In particular, individuals who search using informal methods, such as relying on family and friends, may be more likely to search in places with greater numbers of co-ethnics than they desire. This is because social networks tend to be racially segregated, and so the search information provided by these networks may be less varied, as social capital theorists would predict (Granovetter 1973). More specifically, since social networks tend to be racially and ethnically segregated and community knowledge is shaped by race, those who rely on their network when searching for housing may be more likely (compared to those who use, e.g., newspaper ads or real estate agents) to search in neighborhoods with a greater percentage own group than they might prefer.

The extant theories of residential segregation processes just reviewed have clear expectations about how the housing search process unfolds; importantly, though, these expectations are often implicit and almost always unmeasured. In this paper, we offer a rare and exploratory but systematic look at elements of this process. By assessing the mismatches—and where they occur—we can offer ideas about future data collections and, importantly, policy interventions that might break the cycle of segregation that the outcomes of these searches perpetuate.

## Data and Measures

### Chicago Area Study

We use data from the *2004/2005 Chicago Area Study*, a cross-sectional^3^ survey consisting of a multistage area probability sample (stratified by neighborhood racial/ethnic composition) representative of adults 21 years and older living in households in Cook County, Illinois (which includes the city of Chicago and many surrounding suburbs). In addition to residential preferences, these data include retrospective information on housing searches and the racial/ethnic composition of current neighborhoods. Black and Latino respondents and those living in racially mixed neighborhoods were oversampled. A total of 789 respondents were interviewed face-to-face between August 2004 and August 2005 (response rate: 45 %). For the purpose of our research, we have insufficient numbers of other racial/ethnic groups to do separate analyses, and so we limit our study to white, black, and Latino respondents (*n* = 769 in the total sample, though as noted below our analyses are limited to a smaller subset).

### Racial and Ethnic Neighborhood Preferences

**Fig. 1:**
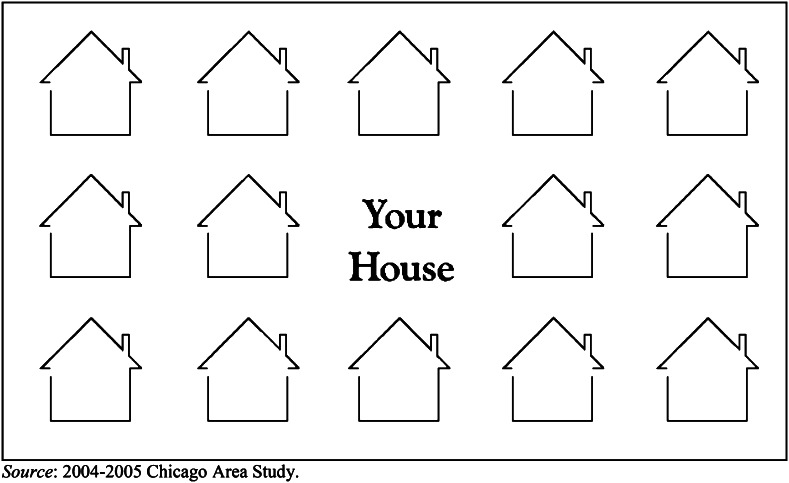
Show card presented to the respondents to indicate the racial/ethnic composition of their ideal neigborhood

Following Charles (2000), to measure racial and ethnic neighborhood preferences respondents were asked to imagine a neighborhood that had the racial/ethnic mix they personally would feel most comfortable living in (the exact question wordings of the main survey questions used in our analyses can be found in the appendix). Next, they were presented a card showing a neighborhood with 15 houses of which the middle house represented their own house (see Fig. 1). Respondents used the letters ‘W’ for white, ‘B’ for black, ‘L’ for Latino, ‘AS’ for Asian American, and ‘AB’ for Arab American, to fill out their ideal neighborhood racial/ethnic composition. We calculated the *percentage of houses that were of the respondent’s own racial/ethnic group*. Respondents who did not complete the housing card were listwise deleted (*n* = 18). To reduce the possibility of socially desirable responses, as much as possible respondents were matched to an interviewer of similar race or ethnicity. In our final sample, 75 % of the respondents were interviewed by someone of their own race.

### Racial/Ethnic Composition of Search and Current Neighborhood

**Fig. 2:**
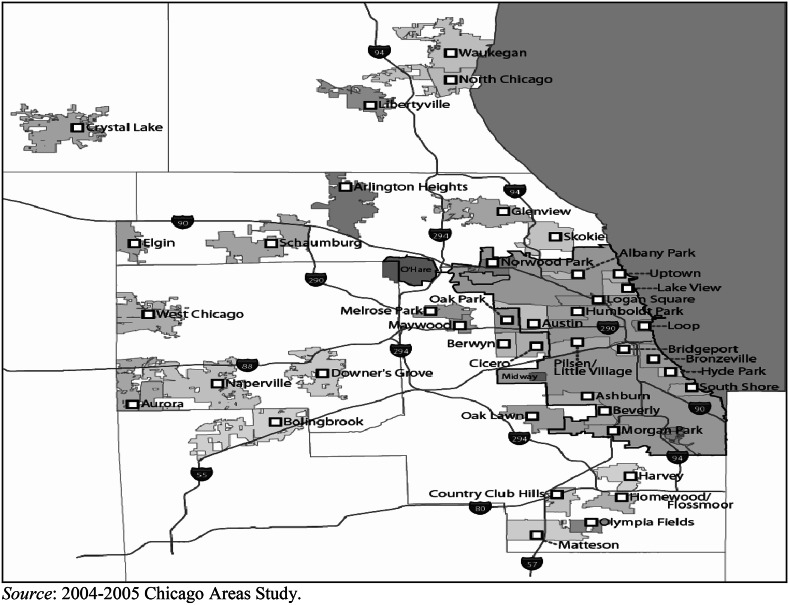
Map provided to the respondents as a tool to indicate their search locations

Respondents were also shown a booklet of maps that labeled 41 communities in the Chicago metropolitan area (see Fig. 2), including neighborhoods in the city of Chicago (*n* = 15 of the total 77) as well as suburban communities (*n* = 26 of the total 288). Inside the city of Chicago, the communities represent administrative boundaries (so-called Communities Areas) that are well known among Chicago area residents, and roughly correspond to ‘neighborhoods.’ Suburban communities are administrative boundaries defined by the census as ‘census designated places’ (CDPs) that usually align with community jurisdiction boundaries. The 41 communities identified on the map were purposively selected to cover substantively important categories (e.g., a range of different racial compositions, different housing prices, locations, etc.) and to be sure that areas would likely be known to a fair number of Chicago residents (i.e., very small communities were excluded). Table 1 compares the racial and ethnic composition of the neighborhoods and communities that were shown on the map (Column 1) with those that were not (Column 2). Communities with populations over 85 % white (“all white” neighborhoods) are underrepresented on the map, whereas racially/ethnically mixed communities are overrepresented compared to their presence in the entire Chicago metropolitan area.

**Table 1:** Racial and ethnic composition of places/communities on the map and overall in the Chicago metropolitan area

	The options people were given to choose from (on the map) (%)	The options people actually have to choose from (in the metropolitan area) (%)
*Racial and ethnic composition* ^a^		
All white	14	44
All black	7	8
All Hispanic	5	1
Mostly white	9	13
Mostly black	7	2
Mostly Hispanic	0	1
Mixed B–W white majority	12	4
Mixed B–W black majority	7	3
Mixed W–H white majority	5	9
Mixed W–H Hispanic majority	5	2
Mixed W–A white majority	2	1
Mixed B–H black majority	0	1
Mixed two groups	7	2
Mixed three groups	21	9
Total	100	100
*n* ^b^	*43*	*365*

*Source* 2000 census

^a^All white: communities where 85 % or more of its residents are white; All black: communities where 85 % or more of its residents are black; All Hispanic: communities where 85 % or more of its residents are Hispanic; Mostly white: communities where 70 % or more of the residents are white and there are fewer than 15 % of any single other racial/ethnic group; Mostly black: communities where 70 % or more of the residents are black and there are fewer than 15 % of any single other racial/ethnic group; Mostly Hispanic: communities where 70 % or more of the residents are Hispanic and there are fewer than 15 % of any single other racial/ethnic group; Mixed black/white with white majority: communities where 51 % or more of the residents are white and more than 15 % are black; Mixed black/white with black majority: communities where 51 % or more of the residents are black and more than 15 % are white; mixed white/Hispanic with white majority: communities where 51 % or more of the residents are white and more than 15 % are Latino; mixed white/Hispanic with Hispanic majority: communities where 51 % or more of the residents are Hispanic and more than 15 % are white; mixed white/Asian with white majority: communities where 51 % or more of the residents are white and more than 15 % are Asian; two-group mixture: communities where there are 40 % or more of two different racial/ethnic groups, but neither is in the majority; Three-group mixture: communities where three racial/ethnic groups have populations in excess of 10 %

^b^The total number of communities represented on the map is 43 because two of the 41 places identified on the map were combinations of two separate communities (Pilsen and Little Village; Homewood and Flossmoor). For calculations of community characteristics shown in this table, these four communities are treated separately

Respondents marked all the identified neighborhoods on the map in which they had searched for housing in the past 10 years. We removed those respondents (*n* = 209) who had lived for more than 10 years at their present address *and* had not actively searched for housing in the meantime. There were also a total of 160 respondents who *did* search for housing in the past 10 years but who indicated that they had not searched for housing in any of the 41 communities shown on the map. These were also removed from our analytic sample, resulting in a final sample size of 382 (123 white, 114 black and 145 Latino respondents).^4^

To indicate the racial/ethnic composition of the search locations we calculated the *average percentage of a respondent’s own racial/ethnic group* in the communities a respondent searched for housing (community figures are based on 2000 census statistics). The racial and ethnic composition of the current neighborhood was measured by the *percentage own racial/ethnic group* in the current neighborhood, based on 2000 census tract statistics.^5^

### Measuring Mismatches

To measure if whites, blacks, and Latinos search for housing and reside in the neighborhoods they say they prefer we calculated the percentage of respondents in each racial/ethnic group that searched and currently reside in neighborhoods with a *similar, larger* or *smaller* percentage of their own racial/ethnic group than in their ideal neighborhood. If the average percentage of own racial/ethnic group in the search locations or in the current neighborhood of a respondent falls within a range of plus or minus 15 % compared to the percentage own group in their ideal neighborhood, we considered preferences and search locations/current neighborhood to be a ‘match.’ Preferences measured using the neighborhood cards are not continuous (they increase in increments of 7 % for each additional house) and, hence, this 15 % difference equals approximately the addition (or subtraction) of two same-race neighbors from a respondent’s neighborhood card. This means that respondents who have a mismatch should have added or removed at least two houses on the preference card to match their average search locations and current neighborhood. Similarly, the percentage of respondents who reside in a neighborhood with a similar, larger, or smaller percentage of own group than in their average search location were calculated.

The variables percentage own racial/ethnic group in respondents’ ideal neighborhood, average search neighborhood, and current neighborhood as well as their associated mismatches are summarized in Table 2. All presented analyses use a weight that incorporates a selection weight and an adjustment for nonresponse (which was the inverse of the response rate in each primary sampling unit). Furthermore, they account for the clustered nature of our sampling approach, using Stata’s “svy” command.

**Table 2:** Descriptive statistics for whites, blacks, and Latinos: range, means/proportion, and standard deviation

	Whites			Blacks			Latinos		
	Range	Mean/*p*	SD	Range	Mean/*p*	SD	Range	Mean/*p*	SD
*Dependent variables*									
Percentage own group in ideal neighborhood	14.28–100	46.49	18.79	0–100	36.76	20.17	0–100	31.71	23.80
Percentage own group in current neighborhood	0–95.97	73.45	15.43	0–100	66.24	36.88	2.68–98.51	50.87	32.76
Average percentage own group in search locations	11.47–89.94	67.61	14.63	1.29–99.00	40.34	22.57	2.33–83.03	32.34	25.62
*Mismatch*									
Percentage own group current neighborhood > ideal neighborhood	0/1	0.68		0/1	0.64		0/1	0.56	
Percentage own group average search location > ideal neighborhood	0/1	0.52		0/1	0.21		0/1	0.29	
Percentage own group current neighborhood > average search locations	0/1	0.24		0/1	0.60		0/1	0.56	
*Explanatory variables*									
Education									
Less than H.S. degree	0/1	0.01		0/1	0.08		0/1	0.36	
H.S. degree	0/1	0.03		0/1	0.25		0/1	0.19	
Some college, less than BA	0/1	0.34		0/1	0.33		0/1	0.35	
BA degree or higher	0/1	0.61		0/1	0.32		0/1	0.10	
Household income									
<$20,000	0/1	0.06		0/1	0.18		0/1	0.30	
$20,000–39,999	0/1	0.08		0/1	0.33		0/1	0.37	
$40,000–79,999	0/1	0.37		0/1	0.31		0/1	0.22	
>$80,000	0/1	0.48		0/1	0.16		0/1	0.09	
General lack of knowledge (# of unknown neighborhoods on map)	0–32	11.53	6.29	0–41	10.99	11.52	0–39	16.56	12.29
Percentage own group in known neighborhood > unknown neighborhoods	0/1	0.84		0/1	0.88		0/1	0.86	0.39
Self-reported housing discrimination	–	–	–	0–3	0.71	1.07	0–3	0.57	1.00
# of years in the Chicago metropolitan area	1–75	27.28	12.07	1–82	31.16	16.97	0–53	17.94	12.51
*n*	123			114			145		

*Source* 2004–2005 Chicago Area Study

## Descriptive Results

We start this section with an overview of the search behaviors of whites, blacks, and Hispanics in the Chicago metropolitan area. The number of indicated search locations ranged from 0 to 41, but on average respondents indicated 3.6 communities on the map in which they had actively searched for housing in the past 10 years. The number of communities in which people reported searching differs among racial and ethnic groups, whereas whites indicated on average 2.8 search communities, Hispanics and blacks generally searched in 4.2 and 4.8 communities, respectively.

The average distance between the communities where respondents said they searched for housing and the respondents’ current neighborhood is 13.3 km. The distance is largest for blacks (17.8 km), followed by whites (12.3 km), and Hispanics (11.4 km). The distance between the average search communities and current neighborhoods is smaller than the average distance between the 41 communities on the map and the current neighborhoods (20.9 km). This suggests that people tend to focus their searches on geographically proximate areas. Due to the cross-sectional and retrospective nature of the data, we cannot make too much of this finding, however, since we do not know where the respondent was living when they undertook the search they are reporting about (it could have been their current neighborhood; it could have been somewhere else altogether).

Table 3 presents an overview of the mean stated racial/ethnic neighborhood preferences, racial/ethnic composition of the search locations, and the composition of the current neighborhood for whites, blacks, and Latinos. First, Panel A shows that all groups drew rather diverse neighborhoods but they also prefer to live in a neighborhood with at least a substantial number of co-ethnics. This is especially true for whites and less so for blacks (*F* = 6.00, *p* < 0.05) and Latinos (*F* = 18.84, *p* < 0.01). Second, all groups currently reside in a neighborhood in which the majority of residents belong to their own racial/ethnic group, though this pattern is stronger for whites (*F* = 20.31, *p* < 0.01) and blacks (*F* = 7.31, *p* < 0.01) than for Latinos (Panel B of Table 3). Panel C shows that whites mainly search in neighborhoods with a substantial proportion of their own group and this is less prominent for blacks (*F* = 48.60, *p* < 0.01) and Latinos (74.5, *p* < 0.01). Blacks and Latinos search in neighborhoods with a substantial proportion of their own group but even larger shares of whites (Panel C of Table 3).

**Table 3:** Stated racial/ethnic preferences, racial/ethnic composition of search locations and current neighborhood

	Whites (*n* = 123)	Blacks (*n* = 114)	Latinos (*n* = 145)
**A. Stated racial/ethnic preferences**			
Mean percentage *whites* in ideal neighborhood	**46.5**	26.7	31.3
Mean percentage *blacks* in ideal neighborhood	15.1	**36.8**	16.1
Mean percentage *Hispanics* in ideal neighborhood	14.9	16.5	**31.7**
**B. Racial/ethnic composition of current neighborhood**			
Mean percentage *whites* in current neighborhood	**73.5**	18.3	38.1
Mean percentage *blacks* in current neighborhood	5.8	**66.2**	5.9
Mean percentage *Hispanics* in current neighborhood	12.1	10.9	**50.8**
**C. Racial/ethnic composition of search locations**			
Mean average percentage *whites* in search locations	**67.6**	44.8	48.8
Mean average percentage *blacks* in search locations	10.1	**40.3**	13.0
Mean average percentage *Hispanics* in search locations	13.9	10.6	**32.3**

Bold values refer to own racial/ethnic group

*Source* 2004–2005 Chicago Area Study

Table 4 identifies the percentage of respondents who have a (mis)match in various combinations of the percentage of their own racial/ethnic group in (1) their ideal neighborhood; (2) their current neighborhood; and (3) their search locations. Panel A reports the extent to which racial/ethnic preferences are reflected in the composition of the neighborhoods where respondents currently live. The majority of white (68 %), black (64 %), and Latino respondents (56 %) live in neighborhoods that have more of their own racial/ethnic group than they say they prefer (statistical tests do not reveal differences across groups on this measure). Panel B of Table 4 shows, furthermore, that the majority of whites in our sample (52 %) search in neighborhoods with a larger share of their own group than they say they prefer. This percentage is significantly larger compared to 21 % of blacks (*F* = 14.2, *p* < 0.01) and 29 % of Latinos (*F* = 6.63, *p* < 0.05). A majority of blacks (62 %) and a plurality of Latinos (47 %) search in neighborhoods that match their preferences with respect to the percentage own group.^6^ Finally, Panel C of Table 4 shows that nearly two-thirds of whites generally reside in neighborhoods that match the percentage own group in the neighborhoods in which they *searched*. The proportion of whites is almost double the approximately one-third of blacks (*F* = 6.27, *p* < 0.05) and Latinos (*F* = 8.59, *p* < 0.01) who live in neighborhoods with similar proportions of own-group residents as the communities in which they searched. Most blacks (60 %) and Latinos (56 %), on the other hand, reside in neighborhoods with a larger share of their own group than in the average neighborhood they searched for housing.

**Table 4:** Percentage (mis)match between percentage own racial/ethnic group in ideal neighborhood, search locations, and current neighborhood by race/ethnicity

	Whites (*n* = 123) (%)	Blacks (*n* = 114) (%)	Latinos (*n* = 145) (%)
**A. Percentage own group in current neighborhood is**			
Similar as in their* ideal* neighborhood (±15 %)	27.2	31.1	30.5
Larger than in their* ideal *neighborhood (>+15 %)	67.6	63.9	56.0
Smaller than in their* ideal* neighborhood (<−15 %)	5.3	5.0	13.6
**B. Average percentage own group in search locations is:**			
Similar as in their* ideal* neighborhood (±15 %)	40.3	61.7	47.1
Larger than in their* ideal* neighborhood (>+15 %)	52.1	20.5	28.8
Smaller than in their* ideal *neighborhood (<−15 %)	7.6	17.8	24.1
**C. Percentage own group in current neighborhood is:**			
Similar as in their* search* locations (±15 %)	63.8	35.6	35.5
Larger than in their* search* locations (>+15 %)	24.4	59.8	56.3
Smaller than in their *search* locations (<−15 %)	11.8	4.7	8.2

*Source* 2004–2005 Chicago Area Study

In sum, whites both seem to search and reside in neighborhoods with a larger percentage own group than they say they prefer. These descriptive results imply that whites experience a mismatch in an early stage of the residential process (i.e., Stage 1); namely, they do not search in neighborhoods that match the racial composition they say they prefer. Meanwhile, blacks and Latinos search in neighborhoods with their preferred percentage of own group, but they live in neighborhoods with larger percentages of their own group than those in which they searched (and those which they preferred). As a result, we conclude that blacks and Latinos experience a mismatch at the later stage of the process (i.e., Stage 2) since they reside in neighborhoods that have both more of their own group than they desire, and also than the neighborhoods in which they searched.

## What Factors are Related to Mismatches?

Our primary aim in this study is to explore the extent to which housing searches match preferences and outcomes; we also laid out a theoretical framework that suggests a range of possible explanations for these mismatches. The preceding group-level analyses suggest that mismatches do occur, and that their patterns are generally consistent with a number of possible explanations. Data constraints—in particular, small sample sizes and a lack of longitudinal data—do not permit a detailed analysis of the individual-level predictors of a mismatch at the two stages. However, our dataset includes a number of variables that allow us to explore the individual-level factors associated with mismatches. We examine who is more likely to have “too many” of their own group by examining its relationship to socioeconomic resources, information resources and experiences of self-reported housing discrimination, variables that correspond to spatial assimilation, place stratification, and information theories.

### Estimating Correlates of Stage One Mismatches Among Whites

In exploring which factors are related to a mismatch, we focus on the most common kind of mismatch within each racial and ethnic group. For whites, we model the Stage 1 mismatch: the probability that whites search in a neighborhood with more whites than in their ideal neighborhood (Table 5). Based on our theoretical framework, the relevant factors are measures of knowledge (i.e., whites might not know about the places that match their preferences) and education (to capture social desirability effects which operate more strongly among the more highly educated). Information resources were measured by a respondent’s knowledge about different neighborhoods in the Chicago metropolitan area that we obtained by asking which neighborhoods on the map (identical to Fig. 2) they did not know anything about. We used the number of neighborhoods a respondent indicated as a measure of a *generallack of knowledge* of the housing market. To assess whether respondents had racially/ethnically biased knowledge of the housing market, we calculated the average percentage of a respondent’s own group in the neighborhoods they indicated they do *not* know anything about and compared this to the average percentage own group in the neighborhoods which they did not indicate (and thus presumably *do* know about). We created a dummy variable identifying those respondents for whom the average percentage own group in the neighborhoods they *do* know is larger than in the ones they do *not* know. We controlled for how many years the respondent has lived in the metropolitan area since people who have lived longer in the area likely know more about the places in it. We measured a respondent’s *education* with four dummy variables: less than high school degree, high school degree, some college but less than a Bachelor’s degree, and Bachelor’s degree or higher (reference).

**Table 5:** Logistic regression analysis of the probability that *whites* (*n* = 123) search in a neighborhood with *more* own group than in their ideal neighborhood; logit coefficients and standard error (SE)

	Coefficient	SE
# of years in the Chicago metropolitan area	−0.001	0.01
General lack of knowledge	0.001	0.03
% own group in known n’hood > unknown n’hood	0.89^†^	0.52
Education: less than BA degree (vs. >BA degree or higher)	0.13	0.78
Intercept	−0.70	0.78

*Source* 2004–2005 Chicago Area Study

^†^ *p* < 0.10; tested two-sided

The results from Table 5 indicate that whites who possess greater knowledge of white neighborhoods than integrated ones search in neighborhoods with more whites compared to their stated ideal neighborhood. White preferences mismatched their search neighborhood 55 % of the time for whites who possessed greater knowledge of white neighborhoods, compared to 33 % of whites with less racially biased knowledge of neighborhoods.^7^ We found no association between education and a mismatch between stated preferences and searches.

### Estimating Stage Two Mismatches Among Blacks and Latinos

We concentrate on the mismatch at Stage 2 for Blacks and Latinos: the probability that blacks and Hispanics reside in a neighborhood with more own group than in the average search location (Table 6). Here the relevant factors are income (i.e., blacks and Latinos might not be able to afford to live in the neighborhoods where they search for housing) and experience with discrimination (i.e., they might face barriers after searching in these neighborhoods). We measured *household income* in four categories: <$20,000; $20,000–39,999; $40,000–79.999; and >$80,000 (reference). Self-reported housing discrimination was measured by three items.^8^ Respondents answered “yes” or “no” to the following questions: (1) whether or not they ever felt they were denied housing by a landlord or real estate agent because of their race/ethnicity, (2) whether or not they ever felt that a real estate agent was showing them only homes in certain neighborhoods because of their race/ethnicity,^9^ and (3) whether or not respondents ever lived in a neighborhood where neighbors made life difficult because of their race/ethnicity. We combined these items into a summary scale of *self*-*reportedhousingdiscrimination*, running from 0 (did not experience any of these) to 3 (experienced all three).

**Table 6:** Logistic regression analysis of the probability that *blacks* (*n* = 114) and *Latinos* (*n* = 145) reside in a neighborhood with *more* own group than in the average search location (model 3); logit coefficients and standard error (SE)

	Blacks		Latinos	
	Coefficient	SE	Coefficient	SE
Income: <$20,000 (vs. >$80,000)	1.70*	0.78	0.68	0.61
Income: $20,000–$39,999 (vs. >$80,000)	−0.48	0.88	−0.34	0.58
Income: $40,000–$79,999 (vs. >$80,000)	−0.95	0.58	N/A^a^	N/A^a^
Self-reported housing discrimination	0.13	0.37	0.11	0.25
Intercept	0.58	0.56	0.13	0.47

*Source* 2004–2005 Chicago Area Study

^a^For Latinos, income categories $40,000–79,999 and >$80,000 are collapsed due to small sample sizes

* *p* < 0.05; tested two-sided

Table 6 shows that blacks in the lowest income category (i.e., <$20,000) are more likely to reside in a neighborhood with more co-ethnics than in the average neighborhood in which they searched for housing. This finding suggests that black residents with low incomes may search in neighborhoods similar to their racial preferences but be unable to afford those neighborhoods, a finding that supports the predictions of the spatial assimilation model.

## Conclusion and Discussion

Past research has examined the association between preferences for particular racial and ethnic neighborhood compositions, and the composition of the current neighborhood. In this paper, we draw on novel data that allow us to provide a preliminary look at how an important intermediate step—the search for housing—influences these relationships. Overall, our results draw attention to the value of examining the search process that precedes the final choice when studying the correspondence between residential preferences and behaviors in particular, but also as an important component to the perpetuation of racial residential segregation.

In line with earlier research that focused only on the differences between residential preferences and current neighborhood racial/ethnic composition without information on the search itself (e.g., Adelman 2005; Clark 1992), we found that residents generally live in neighborhoods with a larger percentage of their own racial/ethnic group than they report preferring. Yet, our study goes beyond these earlier ones because we find interesting racial and ethnic differences in the stage at which these disparities arise, thus emphasizing the value of studying the search process in relation to residential attainment. Our results suggest that whites experience a mismatch between preferences and behavior at an early stage of the residential process: they generally do not search in neighborhoods that match their preferences. In contrast, blacks—and to a lesser extent Latinos—are more likely to search in neighborhoods that more closely correspond to their preferences in terms of their own racial and ethnic group, but when it comes to where they actually live, their neighborhoods have larger percentages of blacks and Latinos, respectively. The greater degree of match for blacks and Latinos in their search locations and preferences suggests that the explanation for the mismatch between where they prefer and where they actually live should be sought in constraints that prevent the final choice from being the one that matches residential preferences rather than in the choice of search destinations in the first place.

Although our data limit the extent to which we can test a range of theoretical explanations for mismatches at an individual level, our analyses does point to some hints about the sources of the mismatches we observe. For whites, we find that the racial blind spots that Krysan and Bader (2009) identified—such that whites are less likely to be aware of racially diverse neighborhoods—may play a role in the mismatch. That is, white Chicago residents with bigger racial blind spots were the significantly more likely to search in communities that had more whites than they said they prefer. Although education did not predict mismatches, which would have been consistent with a social desirability interpretation, this is a rather weak test. Thus, we suggest that it is still possible that the mismatch for whites at Stage 1 is due in part to whites not being as open to integrated neighborhoods as they say they are. Influenced by social desirability, it may be that whites *report* a preference for integrated neighborhoods when in fact they favor predominantly white neighborhoods, contributing to a discrepancy between preferences and residential behaviors. That whites do not search in neighborhoods that match their reported preferences is consistent with this interpretation.

However, there are other possibilities apart from social desirability.^10^ For example, it could be that the relative availability of neighborhoods in the metropolitan area that approximate whites’ racial and ethnic preferences is unfavorable. Whites say they prefer a rather diverse neighborhood as long as whites constitute the largest racial/ethnic group. Although these diverse neighborhoods are overrepresented on the search location map (see Table 1), which would imply we overestimate the chances that there will be a match, we see that whites end up living in predominantly white neighborhoods. So, relatively rare as they are, our maps actually increased the possibility for a match between whites’ ideal diverse neighborhoods and being able to report on our maps that they searched in one. Nevertheless, whites (in contrast to blacks and Latinos) live in the most “available” type of neighborhoods, i.e., predominantly white neighborhoods. This means that the relative availability of neighborhoods could play a role in the mismatch we found.

Furthermore, it may be that people tend to start searching close by their current residence (wherever that might be). If this is the case, and in light of patterns of segregation, for whites, these ‘nearby’ neighborhoods are more likely to be white neighborhoods as well (whereas for blacks and Latinos there is a greater chance that the nearby neighborhoods are more diverse). This spatial dynamic of searches therefore precludes more diversity in whites’ search locations. Additionally, it may be that the ‘daily routines’ of whites may be particularly circumscribed, translating into less exposure to diverse neighborhoods and therefore, a lower likelihood that there is sufficient knowledge to seriously consider diverse neighborhoods as destinations.^11^

With respect to the mismatches of blacks and Latinos, we find some suggestion of the role of spatial assimilation for blacks. That is, blacks with an income of less than $20,000 are more likely to reside in a neighborhood with more blacks than in their search locations. These results also lend indirect support to the place stratification model, since the searches may have been unsuccessful due to discriminatory barriers that discouraged blacks and Latinos from residing in the more integrated neighborhoods that they prefer. Although our self-reported measure of housing discrimination did not reveal significant findings, as we noted, we have no measure of actual discrimination experienced by the searchers.

Our study draws attention to a number of directions for future research that put the spotlight on the search process itself as critical to understanding residential outcomes. For example, all we know from our survey data is that a person searched in a particular neighborhood—we do not know what they learned about the neighborhood or what they experienced in it when they searched. It may be, for example, that neighborhoods that are similar to whites’ preferences for more integrated neighborhoods come with race-related factors that stopped them from even searching in these neighborhoods. Indeed, some scholars have asked whether people really have a preference for a particular racial and ethnic composition, or whether these preferences are driven by socioeconomic concerns instead (Harris 2001). Outcomes of vignette experiments indicate that part of the racial preference patterns found in studies using the show card method can be attributed to neighborhood concerns that are *related* to race, such as poverty, criminality, and bad school quality (Emerson et al. 2001; Krysan et al. 2009; Lewis et al. 2011; St. John and Bates 1990). Nevertheless, the racial and ethnic composition of neighborhoods remained an important and independent factor in shaping residential preferences. In addition, taking into account socioeconomic factors of the neighborhood, Crowder (2000) and Crowder and South (2008) demonstrate that the racial and ethnic neighborhood composition is still an important determinant of whites’ residential mobility. Understanding the manner by which whites determine where to search—and the role of race/ethnicity versus social class—is of great importance.

Overall, in examining the racial and ethnic composition of neighborhoods in which people searched for housing, we seek to highlight the potential role that housing searches play in contributing to the discrepancy between residential preferences and current neighborhood racial and ethnic composition, in particular. More broadly, however, our goal is to bring systematic attention to the role of housing searches to the residential attainment processes and their stratified outcomes. Our data have several limitations, but these suggestive results draw attention to the need to better understand the housing search process and call for the identification of data collection and research design strategies that can address them and provide a more decisive answer to the broader theoretical frameworks that underlie our analyses.

In interpreting our results, several of these shortcomings should be kept in mind. First, we used a non-random sample of 41 communities on our map of the Chicago metropolitan area. For those who prefer white neighborhoods, the proportion of neighborhoods on the map that match their preferences is smaller than they in fact are. Similarly, for those who prefer racially and ethnically mixed neighborhoods, the proportion of neighborhoods on the map is larger than they would encounter in Chicago as a whole. It is hard to determine what consequences of imperfectly measuring the relative racial and ethnic distribution of neighborhoods has for our observed mismatches. Yet, given that a majority of respondents say they prefer very mixed areas, we believe that it most likely reduced mismatches because respondents had more opportunities among the 41 to choose their ideal (which many said was racially mixed).

Second, the use of cross-sectional data means that we cannot draw causal inferences. With respect to the correlate of mismatches, for example, we have measured income at the time of the survey, not at the time of the search; it is very possible that economic constraints were very different at the two times. In addition, with respect to our key measures of interest, information on preferences and current neighborhood were measured at the same time, and information on respondents’ searches (though not their reporting of them) predated statements of preferences. Because of this, we cannot rule out the possibility that residents adjusted their preferences based on their search experiences. Similarly, not only searches, but also people’s current neighborhood might have informed racial residential preferences as they were measured simultaneously in time. This might have affected our results in two ways: it might have either underestimated mismatches in the case of people who could not realize their initial preference but adjusted their attitudes to the situation in the current neighborhood (or based on their search experiences) or overestimated mismatches for those residents who search or reside in a neighborhood that matched their original preferences but—for whatever reason—change their preferences.

Thus, we clearly do not have the appropriate temporal order of preference, search, and move data. We ask respondents to indicate their current preferences, fully aware that their past searches and current residences might influence their current racial preferences. Future research should prospectively investigate residential preferences and where people move. Such a study is obviously complicated by the fact that the very nature of moving makes following respondents difficult; the payoff, however, would be clear insight into an area potentially ripe for intervention—encouraging residents to search in neighborhoods with their stated level of diversity even if it is not on their initial list of search destinations.

This focus on housing searches not only has value in terms of social science debates and understanding of the causes of residential segregation as well as neighborhood selection processes that relate to a range of ‘neighborhood effects’ research, but also there are significant policy implications as well. Indeed, the U.S. Department of Housing and Urban Development recently issued a 5-year Research Roadmap (Office of Policy Development and Research 2013), citing one of HUD’s four programmatic goals to “Build inclusive and sustainable communities free from discrimination.” The Research Roadmap identified a number of top priority research projects to accomplish this goal, including one that examined the housing search process of racial and ethnic minorities. Understanding this process was identified as foundational for a number of core HUD programs and policies, including the Housing Choice Voucher program, housing integration strategies, and discrimination testing and enforcement. As the Roadmap notes,

…HUD does not know how households search for housing and what their preferences are when searching for housing. This research will shed light on how housing search and preference affect HUD’s fair housing/Affirmative Furthering Fair Housing goals and how they promote or deter goals to build inclusive communities (2013, p. 98).

Our research contributes to this policy debate, suggests next steps, and reinforces its importance.

The present study is, therefore, a first step in pointing out the mismatch between stated preferences, search locations, and outcomes. The results compel future research to gather new large-scale longitudinal data in which residential preferences and complete housing search and neighborhood careers can be followed over time to test our causal implications and to take into account the full set of possible residential destinations. It is also important for future studies to link these housing careers to information about individual socioeconomic resources, housing market knowledge and local discrimination to understand more thoroughly the relevance of the housing search for interpreting spatial assimilation, place stratification, and information models. Using an innovative dataset that measured for the first time the search locations of residents of a major metropolitan area, we have drawn attention to the possibility of the mismatch between stated preferences, search locations, and outcomes in general, and the importance of examining more carefully the search process in particular if we hope to better understand the way in which racial residential segregation is perpetuated—or the possibilities for helping to break it down.

## Appendix

See Table 7.

**Table 7:** Exact question wording of main (non-demographic) survey questions

Variables/predictors	Exact question wording
1. Racial/ethnic composition of ideal neighborhood (racial/ethnic neighborhood preferences)	Now I would like you to imagine an ideal neighborhood that had the ethnic and racial mix you personally would feel most comfortable in. Here is a card that shows a neighborhood with several houses in it. Using the letters W for Whites, B for Black, H for Hispanic, As for Asian American and AB for Arab American, please put a letter in each of these houses to represent your ideal neighborhood where you would most like to live.
2. Racial/ethnic composition of search locations	This is a booklet of maps of the Chicago metropolitan area. The map shows major roads and several communities as well as specific areas in the city of Chicago. For each of the questions I ask, please mark all of the communities or towns that fit your answer. On the third map, mark the communities where you have searched for a house or apartment in the past 10 years
3. Knowledge about different neighborhoods in the Chicago metropolitan area	On this first map, please mark any communities that you don’t know anything about
4. Reported housing discrimination	Now I would like you to ask about several experiences you might have had when looking for housing
	Have you ever felt that you were denied housing because a landlord or real estate agent didn’t want to sell or rent to you because of your race or ethnicity?
	Have you ever felt that a real estate agent was showing you only homes in certain neighborhoods because of your race or ethnicity?
	Have you ever lived in a neighborhood where neighbors made life difficult for you or your family because of your race or ethnicity?

*Source* 2004–2005 Chicago Area Study

## Footnotes

1 Note that this emphasis on housing searches can shed light on how racial residential segregation is perpetuated—or how it can be reduced—and it can also inform the lively debate throughout social sciences on the issue of neighborhood effects. Specifically, there is a thorny selection problem in any studies trying to understand how one’s neighborhood impacts a range of outcomes, including health for example. The question is whether the neighborhood impacts one’s health, for example, or whether people who are unhealthy “select” a certain kind of neighborhood. By focusing on the search process, and understanding how people end up living where they do, we can inform this debate (Krysan et al. 2014).

2 As noted above, an alternative to these substantive explanations for the mismatch is a methodological one: individuals may state a preference for more diversity in their neighborhoods than they actually desire due to social desirability pressures to report more liberal racial attitudes in a survey interview. Existing research suggests this bias is stronger for whites than African Americans; little is known about its influence on Latinos (Krysan 1998; Krysan & Couper 2003). In all cases, however, we expect social desirability pressures to be strongest for the measure of residential preferences, and so its contribution to possible mismatches is greater at stage one of the process, where individuals do not search in neighborhoods that match their preferences in the first place because those preferences for diversity are over-stated.

3 The cross-sectional nature of our data bears special mention since we measure preferences, search locations, and outcomes all at a single point in time. Therefore, issues of causal direction remain uncertain as preferences might not only influence the choice for a particular neighborhood, but experiences during the search process and the racial/ethnic composition of the current neighborhood might also affect people’s preferences. Our data do not allow us to address this problem of reverse causality. Instead, our goal is to illuminate the degree to which preferences differ from searches and current neighborhood conditions and the individual factors associated with possible mismatches. As we note in the conclusion of the paper, we fully acknowledge the limitations of our data, but we argue that it is the best we have and is suitable for our purposes of illuminating this understudied element of segregation processes.

4 Additional analyses indicated that in the removed subsample (i.e., those who did not search for housing in the past 10 years and those who did not search in one of the 41 communities), whites, blacks, and older people are slightly overrepresented. Furthermore, the omitted respondents are slightly more likely to have an own group neighborhood preference and to live in a neighborhood with larger shares of their own group. Nevertheless, patterns of mismatches between preferences and current neighborhoods are similar to the ones found in the analytic sample.

5 We acknowledge that the variation in the geographical scale of the neighborhoods in the various measurements could in itself create a mismatch in racial and ethnic composition. The show card used for the “ideal” neighborhood implies a small area (See Fig. 1), whereas the “current” and “searched” neighborhood measures use larger census tracts and communities. Mismatches might arise either because individuals may attach particular importance to the racial/ethnic composition of their *immediate* (micro) neighborhood (rather than the larger community), or because individuals live in a micro neighborhood that matches their preferences but that is located in a larger community that does not match. Yet, we believe that this possible bias is similar for whites, blacks, and Latinos. It seems, therefore, unlikely that differences in the geographic scale of neighborhoods across our measurement instruments could account for racial/ethnic differences in the degree of mismatch.

6 Additional analyses in which we calculated if *at least one* search location (instead of the average location) matched their ideal neighborhood, showed a similar pattern. Blacks (and to a smaller extent Latinos) search more often than whites in at least one neighborhood that matches their preferences in terms of percentage own group.

7 We calculated the value of racially biased knowledge as P(mismatch) = exp(*β*_0_ + *β*_biased knowledge_)/[1 + exp(*β*_0_ + *β*_biased knowledge_) = exp(.19)/[1 + exp(.19)] = .55 and for non-racially biased knowledge as P(mismatch) = exp(*β*_0_)/[1 + exp(*β*_0_) = exp(−.70)/[1 + exp(−.70)] = .33.

8 Self-reported housing discrimination is an imperfect measure of *actual* discrimination in the housing market. Yet, in the absence of data on actual discrimination practices, we consider it a reasonable proxy.

9 If respondents never used a real estate agent we considered them as not having experienced discrimination on this item.

10 We gratefully acknowledge the anonymous reviewer who pointed out these alternative explanations.

11 This might imply an interaction between ‘general lack of knowledge’ and “composition of the neighborhoods about which the respondent claims little knowledge” as a predictor of the mismatch between preferences and search locations. In separate analyses (not shown) we included this as a predictor but it was not statistically significant. It may be that the small sample sizes are responsible for this null finding.
